# Hypoxia‐induced HIF‐1α and ZEB1 are critical for the malignant transformation of ameloblastoma via TGF‐β‐dependent EMT

**DOI:** 10.1002/cam4.2667

**Published:** 2019-11-01

**Authors:** Shohei Yoshimoto, Fumie Tanaka, Hiromitsu Morita, Akimitsu Hiraki, Shuichi Hashimoto

**Affiliations:** ^1^ Section of Pathology Division of Biomedical Sciences Department of Morphological Biology Fukuoka Dental College Fukuoka Japan; ^2^ Division of Oral and Medical Management Department of Oral and Maxillofacial Surgery Fukuoka Dental College Fukuoka Japan; ^3^ Department of General Dentistry Fukuoka Dental College Fukuoka Japan

**Keywords:** ameloblastoma, carcinogenesis, epithelial‐mesenchymal transition, odontogenic tumors, transforming growth factor beta

## Abstract

Ameloblastic carcinoma (AC) is defined as a rare primary epithelial odontogenic malignant neoplasm and the malignant counterpart of benign epithelial odontogenic tumor of ameloblastoma (AB) by the WHO classification. AC develops pulmonary metastasis in about one third of the patients and reveals a poor prognosis. However, the mechanisms of AC oncogenesis remain unclear. In this report, we aimed to clarify the mechanisms of malignant transformation of AB or AC carcinogenesis. The relatively important genes in the malignant transformation of AB were screened by DNA microarray analysis, and the expression and localization of related proteins were examined by immunohistochemistry using samples of AB and secondary AC. Two genes of hypoxia‐inducible factor 1 alpha subunit (*HIF1A*) and zinc finger E‐box‐binding homeobox 1 (*ZEB1*) were significantly and relatively upregulated in AC than in AB. Both genes were closely related in hypoxia and epithelial‐mesenchymal transition (EMT). In addition, expressions of HIF‐1α and ZEB1 proteins were significantly stronger in AC than in AB. In the cell assays using ameloblastoma cell line, AM‐1, hypoxia condition upregulated the expression of transforming growth factor‐β (TGF‐β) and induced EMT. Furthermore, the hypoxia‐induced morphological change and cell migration ability were inhibited by an antiallergic medicine tranilast. Finally, we concluded that hypoxia‐induced HIF‐1α and ZEB1 were critical for the malignant transformation of AB via TGF‐β‐dependent EMT. Then, both HIF‐1α and ZEB1 could be potential biomarkers to predict the malignant transformation of AB.

## Graphical abstract

We aimed to clarify the mechanisms of malignant transformation of ameloblastoma or ameloblastic carcinoma carcinogenesis. We found that hypoxia‐induced HIF‐1a and ZEB1 are critical for the malignant transformation of ameloblastoma via TGF‐beta‐dependent EMT.

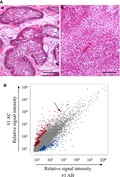

## 1 INTRODUCTION

Ameloblastoma (AB) is worldwide the most common epithelial odontogenic benign neoplasm. AB usually occurs in the mandibular or maxillary bones and shows slow but sometimes locally invasive growth with bone absorption. Because of its locally invasive growth, surgical resection is selected for the main treatment of AB. Ameloblastic carcinoma (AC) is defined as a rare primary epithelial odontogenic malignant neoplasm and a malignant counterpart of AB by the WHO classification.^1^ Clinical behavior of AC is more aggressive and invasive than that of AB. AC sometimes shows recurrence and metastasis and has a poor prognosis.^2^, ^3^ Most AC arose de novo, but some arose in the preexisting AB.^4^, ^5^ However, the mechanisms of AC oncogenesis have not yet been clarified.

Tumor development usually depends on the surrounding environment, known as tumor microenvironment.^6^, ^7^ When the tumor grows rapidly, the microenvironment often causes local ischemia and hypoxia. This hypoxic state plays some crucial roles in the tumor development. In this condition, hypoxia‐inducible factor 1α (HIF‐1α) that is a transcription factor and is known as a molecular sensor of oxygen tension plays many roles for tumor cells to adapt low oxygen levels. In the normoxic condition, HIF‐1α is degraded by 26S proteasome through hydroxylation and ubiquitination. Under the hypoxic condition, expression levels of HIF‐1α are elevated because of its decreased degradation by proteasome.^8^ In some previous studies, HIF‐1α induces epithelial–mesenchymal transition (EMT) of tumor cells in hepatocellular carcinoma.^9^, ^10^ EMT plays essential roles in tumor invasion and metastasis.^11^, ^12^ EMT has been originally known as a phenotypic change during embryonic development, tissue remodeling, and wound healing.^13^ When EMT occurs, cells lose intercellular adhesion, alter morphology to spindle‐shaped appearances, and increase mobility.^13^

In this study, we screened significantly and relatively up‐ or downregulated genes in the malignant transformation of AB by DNA microarray analysis and focused on two upregulated genes, hypoxia‐inducible factor 1, alpha subunit (*HIF1A*), and zinc finger E‐box‐binding homeobox 1 (*ZEB1*) genes. These two genes both play an important role in the EMT induction. Results of the histopathological and in vitro hypoxic cell assay studies clarified that hypoxia‐induced HIF‐1α and ZEB1 were critical for the malignant transformation of AB via TGF‐β‐dependent EMT.

## 2 MATERIALS AND METHODS

### 2.1 Patients and clinicopathological profiles

This clinical study using the patients' information was done under the permission of the ethics committee in Fukuoka Dental College (ID number: 339). Eleven cases of AB (male/female: 8/3, mean age: 35.8 years old (range: 15‐66 years old)) and 5 cases of AC (male/female: 3/2, mean age: 44.4 years old (range: 16‐72 years old)) were examined. These Japanese patients underwent surgery at Fukuoka Dental College Hospital, Fukuoka, Japan, between 2010 and 2018. Neither chemotherapy nor irradiation was prescribed for the patients before surgery. The histological classification was performed according to the criteria of the “2017 WHO Classification of Head and Neck Tumours.”^1^ The clinicopathological profiles of the patients are summarized in Table 1. For more details, in the five #1 to #5 AC cases in our diagnostic archives, #1 and #2 ACs occurred in the preexisting #1 and #2 ABs, respectively, and #3 AC contained both AB and AC components when it was examined, thus #1 to #3 AC cases were diagnosed as secondary ACs.

**Table 1:** Summary of the clinicopathological characteristics of ameloblastoma (AB) and ameloblastic carcinoma (AC) patients examined

AB	Age	Sex	Site	Type	AC	Age	Sex	Site	Type
#1	21	F	Mandible	Follicular	#1	21	F	Mandible	Secondary of #1 AB
#2	15	M	Mandible	Plexiform	#2	16	M	Mandible	Secondary of #2 AB
#3	49	M	Mandible	Plexiform	#3	58	F	Maxilla	Secondary
#4	66	M	Maxilla	Follicular	#4	55	M	Mandible	Primary
#5	36	M	Mandible	Follicular	#5	72	M	Mandible	Primary
#6	48	F	Mandible	Plexiform					
#7	24	M	Mandible	Plexiform					
#8	54	F	Mandible	Plexiform					
#9	28	M	Mandible	Follicular					
#10	33	M	Mandible	Follicular					
#11	20	M	Mandible	Follicular					

### 2.2 Total RNA isolation from formalin‐fixed paraffin‐embedded samples

According to the manufacture's protocols, total RNA was isolated from formalin‐fixed paraffin‐embedded (FFPE) samples using ReliaPrep FFPE Total RNA Miniprep System (Promega Corp). The quality of the RNA was checked using Agilent 2200 TapeStation (Agilent Technologies).

### 2.3 Gene expression microarrays

According to the manufacture's protocols, the cRNA was amplified and labeled using the GeneChip WT Pico Reagent Kit (Thermo Fisher Scientific) and hybridized using Clariom D Array, Human (Thermo Fisher Scientific). All hybridized microarray slides were scanned using an Affymetrix GeneChip Scanner. Relative hybridization intensities and background hybridization values were calculated and normalized using Affymetrix Expression Consol Software (ver. 1.4.1).

### 2.4 Antibodies

The primary antibodies, rabbit anti‐human E‐cadherin monoclonal antibody (#3195) and rabbit anti‐human β‐actin monoclonal antibody (#4967), were purchased from Cell Signaling Technology Inc. Rabbit anti‐human ZEB1 antibody (HPA027524) was purchased from Sigma‐Aldrich. Mouse anti‐human HIF‐1α monoclonal antibody (ab16066), rabbit anti‐human TGF‐β antibody (ab92486), and mouse anti‐vimentin antibody (ab8978) were purchased from abcam. The secondary antibodies, horseradish peroxidase (HRP)‐conjugated polymer anti‐rabbit and anti‐mouse antibodies, were purchased from DAKO‐Agilent Technologies Co. HRP‐linked anti‐rabbit and ‐mouse antibodies were purchased from Cell Signaling Technology Inc. Alexa Fluor 594‐conjugated goat anti‐rabbit IgG and Alexa Fluor 488‐conjugated goat anti‐mouse IgG antibodies were purchased from Thermo Fisher Scientific.

### 2.5 Immunostaining for tissues and cells

10% neutral buffered formalin‐fixed and paraffin‐embedded tissue blocks were cut into 4 μm‐thick sections for HE and immunohistochemical staining. Antigen retrieval was performed for all sections by an autoclave treatment at 121°C for 5 minutes in 0.01 mol/L citrate buffer, pH 6.0. Immunostaining was performed by using EnVision/ horseradish peroxidase (HRP) kit (DAKO‐Agilent Technologies Co., Santa Clara, CA, USA). Briefly, the sections were treated with a 0.1% hydrogen peroxide‐methanol solution to inhibit endogenous peroxidase activity and a 5% BSA/TBS to block any non‐specific binding of primary antibodies. Subsequently, each section was incubated with the primary antibody against HIF‐1α (1:100 dilution), ZEB1 (1:500 dilution), or E‐cadherin (1:200 dilution) at 4°C overnight. These sections were then incubated with HRP‐conjugated polymer anti‐rabbit or anti‐mouse antibody. The peroxidase activity was visualized using 0.1% 3, 3′‐diaminobenzidine and 0.01% hydrogen peroxide in TBS. For the immunofluorescent staining, after incubation with each primary antibody, the section was incubated with Alexa Fluor 594‐conjugated goat anti‐rabbit IgG (1:1000 dilution) or Alexa Fluor 488‐conjugated goat anti‐mouse IgG (1:1000 dilution) secondary antibody, followed by nuclear counterstaining with DAPI (1:3000 dilution). Then, sections were mounted using VECTASHIELD (Vector Lab.). The images of HE and immunohistochemical staining were captured using microscope (AXIO Vert.A1, Carl Zeiss Inc). The images of immunofluorescent staining were visualized and captured at the appropriate wavelength using a fluorescence microscope (LSM 710, Carl Zeiss Inc). The images were processed using a ZEN 2010B Sp1 Ver. 6.0.0.485 software (Carl Zeiss Inc.). For immunocytochemistry, the same immunostaining procedure described above was applied for cells after fixation with 4% paraformaldehyde (PFA).

### 2.6 Immunohistochemical assessment

The degree of positivity of immunoreaction in each lesion was determined according to the method modified from the original one described by Allred et al.^14^ Briefly, we randomly chose three areas at the lesion of AB or AC section and counted the number of tumor cells immunoreactive for HIF‐1α or ZEB1 in their cytoplasm and/or nuclei. The percentage of immunoreactive atypical cells was described as proportion score (PS) [scored on a scale of 0‐3; 0:0%, 1: less than 10%, 2: less than 30%, 3: equal to or more than 30%]. Staining intensity was also described as intensity score (IS) (scored on a scale of 0‐3; 0: negative, 1: weakly positive, 2: intermediately positive, 3: strongly positive). The proportion and intensity scores were summed to produce total score (TS = PS + IS) [scored on a scale of 0, 2‐6]. Then, the mean score of TS was statistically compared for analyzing the correlation between HIF‐1α or ZEB1 expression and histopathological features.

### 2.7 Cell culture

The human ameloblastoma cell line AM‐1 was established from a plexiform‐type ameloblastoma representing typical features of native cells^15^ and was kindly donated by Dr Mitsuyasu (Kyushu University).^16^ Cells were grown in defined keratinocyte serum‐free medium (D‐KSFM; Invitrogen) and incubated at 37°C, 5% CO₂. Cells were exposed to hypoxia (1.0% O2) in a hypoxic chamber (Anaelopack Kenki; Mitsubisi Gas Chemical Company) for the indicated time period. EMT induction was performed as previously described.^17^ Briefly, cells were treated with TGF‐β (5 ng/mL) in the MEM supplemented with EGF (10 ng/mL), 100× insulin‐transferring selenium, and 50 nmol/L hydro‐cortisone, for 72 hours.

### 2.8 Western blotting

AM‐1 cells were homogenized in an ice‐cold lysis buffer and centrifuged for 30 minutes at 4°C. The supernatants (20 μg) were separated on a 4%‐12% Bis‐Tris Plus gel (Thermo Fisher Scientific) and transferred to a polyvinyldifluoride membrane (Millipore). Immunoblot analyses were performed using mouse anti‐HIF‐1α, rabbit anti‐ZEB1, rabbit anti‐human E‐cadherin, mouse anti‐vimentin, and rabbit anti‐human TGF‐β antibodies (all 1:1000 dilution). Rabbit anti‐human β‐actin antibody (1:5000 dilution) was used as an internal standard. Blots were developed with horseradish peroxidase (HRP)‐linked secondary antibodies (1:3000 dilution) and visualized by the enhanced chemiluminescence (ECL) system using ImmunoStar Zeta (Wako), and the bands were detected by LAS‐4000 (GE Healthcare).

### 2.9 Wound healing assay

Wounds were prepared by using Culture‐Insert (2 well; ibidi). AM‐1 cells were cultured for 48 hours in an objective condition and removed the Culture‐Insert. After 24 hours sustained culture, the area of remaining wounds was determined using ImageJ (National Institutes of Health). The closing ratio (CR) of the wounded area was calculated by the following formula: CR = (w − rw)/w × 100 (%) (w: wounded area at the start point, rw: remaining wounded area). Then, the mean value of the CR in each condition was statistically compared.

### 2.10 Statistical analysis

All data were expressed as the mean ± standard error of the mean (SEM). Student's *t* test and Mann‐Whitney U test were applied for the comparison between two groups. Kruskal‐Wallis test and consecutive Mann‐Whitney *U* test with a Bonferroni correction were applied for multiple comparisons. Statistical significance was set as *P < .05, **P < .01 and ***P < .001.

## 3 RESULTS

### 3.1 *HIF‐1α* and *ZEB1* genes were representatively and significantly upregulated in AC by DNA microarray analysis

**Figure 1:**
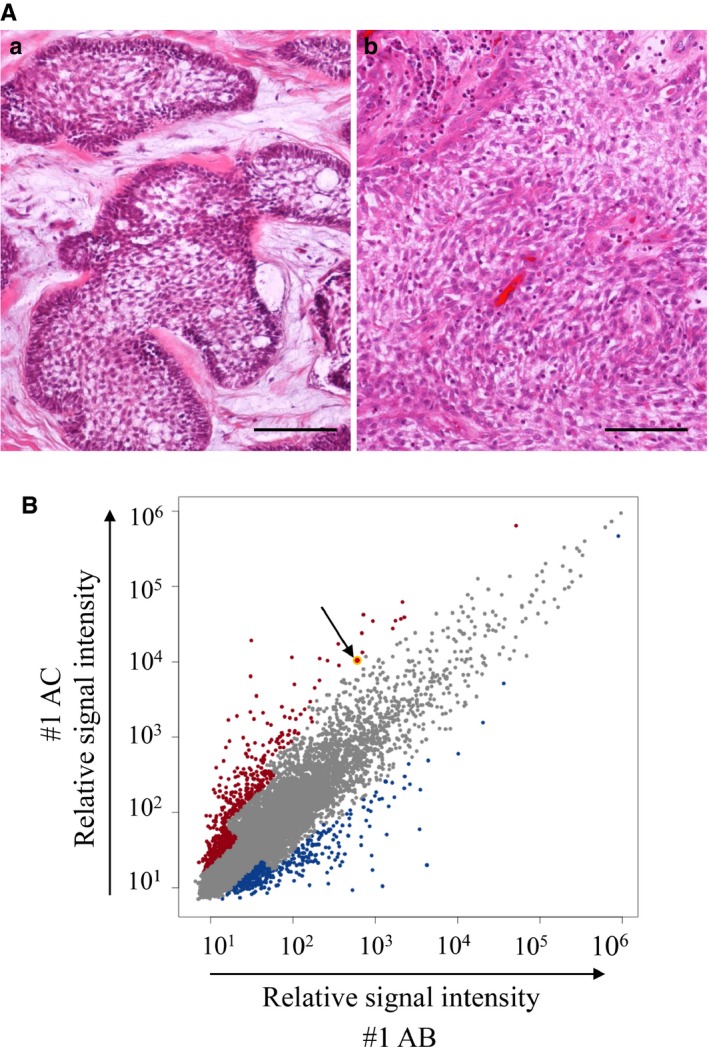
Histological feature and gene expression analyses in AB and AC. A, HE staining of #1AB (a) and #1AC (b). Tumor cell nests reveal peripheral columnar cell palisading and loosely arranged central stellate cell feature (a). The tumor cells reveal severe cellular atypia such as large and hyperchromatic nuclei and prominent nucleoli (b). Scale bars: 100 μm. B, Scatterplots representing the expression of genes in #1AB and #1AC *x*‐axis indicates normalized log_2_ signal intensity of #1AB, and *y*‐axis indicates normalized log_2_ signal intensity of #1AC. Blue and red dots indicate genes which are upregulated in #1AB and in #1AC, respectively. The yellow dot pointed by an arrow indicates *HIF1A* gene expression

As described in materials and methods, the three #1 to #3 AC cases were diagnosed as secondary ACs (Table 1). In these three secondary AC cases, #1 AC and the preexisting #1 AB obtained from the same patient showed typical AC and AB histopathological features, respectively. Namely #1 AB showed follicular growth of two types of tumor cells consisting of peripheral columnar palisading cells and loosely arranged central stellate cells (Figure 1A; a), and #1 AC consisted of solid growth of severely atypical odontogenic tumor cells partly showing AB‐like morphology or spindle‐shaped appearance suggesting EMT induction (Figure 1A; b). Thus, we chose #1 AC and the preexisting #1 AB materials and total RNA obtained from the samples were applied for the DNA microarray analysis to screen the significantly and relatively important genes in the malignant transformation of AB. We investigated AB malignant transformation‐related genes using gene ontology (GO) terms. Although many genes were upregulated in #1 AC, we first focused on one GO term, “transcription factor activity, sequence‐specific DNA binding” and picked *HIF1A* up as one of the most important genes in our aim with the result that *HIF1A* was actually the third upregulated gene in #1 AC (Table 2). Scatterplots representing the expression of genes in #1 AB and #1 AC also showed that *HIF1A* was significantly increased in #1 AC (Figure 1B; yellow circle indicated by an arrow). From a different point of view, as #1 AC histophathologically showed spindle‐shaped appearance of tumor cells suggesting EMT induction, we also focused on EMT‐related transcription factors. As with the previous reports that several EMT‐related transcription factors were upregulated in the EMT induction in cancer developments, in this #1 AC, *ZEB1* was the most and significantly upregulated gene by the DNA microarray analysis in the EMT‐related transcription factors (Table 3). Moreover, *ZEB1* was also the tenth highest gene in the upregulated genes with the GO term ID (Table 2). From these results, we hypothesized that hypoxia‐induced HIF‐1α and ZEB1 played some critical roles in the malignant transformation of AB via EMT induction.

**Table 2:** Ten most upregulated genes from the 1175 genes with a GO term ID (GO: 0003700/transcription factor activity, sequence‐specific DNA binding)

Gene symbol	*Z* score	Fold changes #1 AB vs #1 AC	Gene name
PRDM1	4.579540676	102.8520686	PR domain containing 1, with ZNF domain
TSC22D3	3.40930138	34.41627375	TSC22 domain family, member 3
HIF1A	2.026398792	17.01183663	Hypoxia‐inducible factor 1, alpha subunit
RCAN1	3.586628078	14.59752625	Regulator of calcineurin 1
MEIS2	3.218579318	11.278482	Meis homeobox 2
TFEC	3.001384157	9.68589256	Transcription factor EC
BHLHE41	1.559432856	9.652883719	Basic helix‐loop‐helix family, member e41
TSC22D1	1.535772787	9.382549523	TSC22 domain family, member 1
KLF5	2.940451309	9.280953297	Kruppel‐like factor 5 (intestinal)
ZEB1	1.947696648	8.768748792	Zinc finger E‐box binding homeobox 1

Abbreviations: AB, Ameloblastoma; AC, Ameloblastic carcinoma.

**Table 3:** Fold changes of EMT‐related transcription factors expression

Gene symbol	Fold changes #1 AB vs #1 AC	Gene name
ZEB1	8.768	Zinc finger E‐box binding homeobox 1
SNAI1	1.105	Snail family zinc finger 1
SNAI2	1.090	Snail family zinc finger 2
TWIST1	0.932	Twist family bHLH transcription factor 1
ZEB2	0.899	Zinc finger E‐box binding homeobox 2
TWIST2	0.704	Twist family bHLH transcription factor 2

Abbreviations: AB, Ameloblastoma; AC, Ameloblastic carcinoma.

### 3.2 Immunohistochemical expressions of HIF‐1α and ZEB1 were stronger in AC than in AB

**Figure 2:**
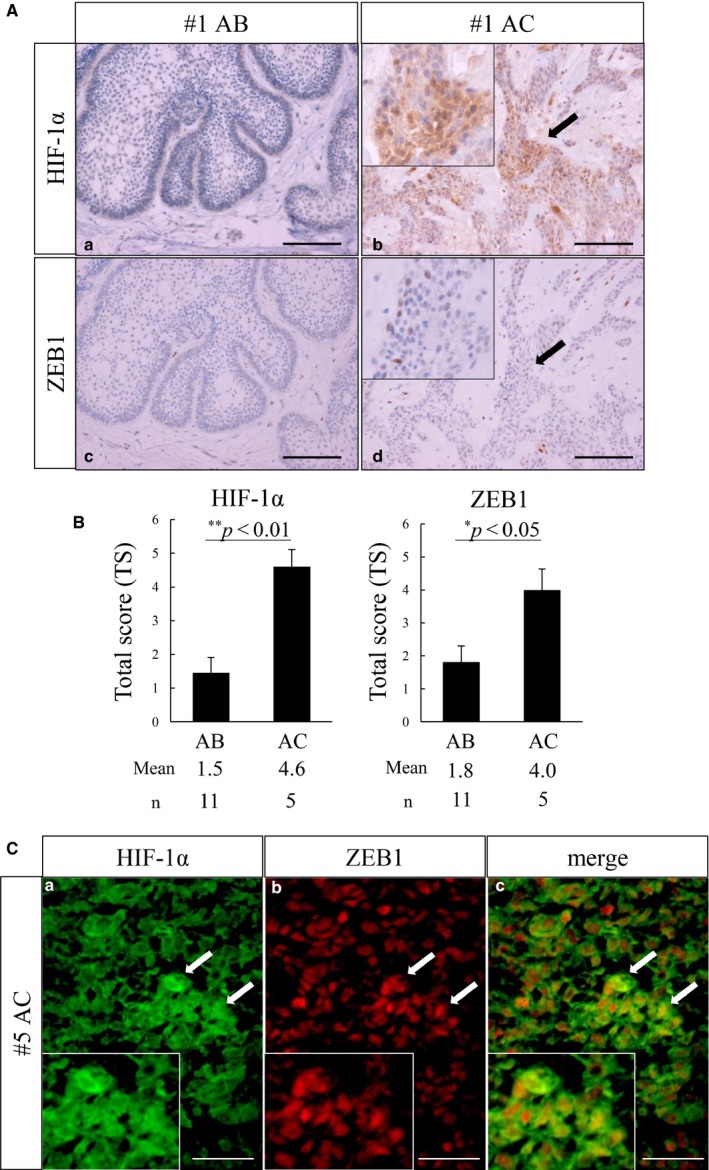
Immunohistochemical analyses of HIF‐1α and ZEB1 expressions in AB and AC. A, HIF‐1α expression is shown in #1AB (a) and #1AC (b). HIF‐1α immunoreaction is weak in AB (a), but apparent in AC (b; arrow and inset). ZEB1 expression is shown in #1AB (c) and #1AC (d). ZEB1 immunoreaction is weak in AB (c), but apparent in the nuclei of AC (d, arrow and inset). Scale bars: 100 μm. B, Comparison analyses of total scores (TSs) of HIF‐1α or ZEB1 expression in between AB and AC. TSs of HIF‐1α and ZEB1 are both significantly increased in AC. Statistical significance was set as *P < .05 and **P < .01. C, Dual immunocytostaining of HIF‐1α (a; green) and ZEB1 (b; red) in #5AC. Co‐expression of HIF‐1α and ZEB1 is observed in tumor cells (arrows in a, b, c). Merged image: (c). Scale bars: 100 μm

We examined the cellular localization and total scores (TSs) of HIF‐1α and ZEB1 protein expressions in all eleven AB and five AC cases described in Table 1. Immunohistochemically, HIF‐1α expression was weak in ABs (Figure 2A; a) but apparent in both the nucleus and cytoplasm of AC cells (Figure 2A; b, arrow and inset). Other AB and AC cases also revealed the same immunohistochemical tendency (Supporting Information 1). ZEB1 expression was also weak or not apparent in ABs (Figure 2A; c) but was distinct and scattered in the nuclei of AC cells (Figure 2A; d, arrow and inset). In the correlation of the mean values of HIF‐1α or ZEB1 TSs in between ABs and ACs, both expressions in ACs were statistically and significantly higher than those in ABs (Figure 2B). Furthermore, co‐expression of HIF‐1α and ZEB1 in the nuclei of AC cells tended to be observed in the regions showing prominent invasive or aggressive growth of AC cells with a loss of cell‐cell adhesion or individual growth appearances (Figure 2C, arrows). From these findings, both HIF‐1α and ZEB1 might contribute to and be predictive potential biomarkers for the malignant transformation of AB.

### 3.3 Hypoxia‐induced HIF‐1α and ZEB1 expressions and EMT‐like morphological changes in AM‐1 cells

**Figure 3:**
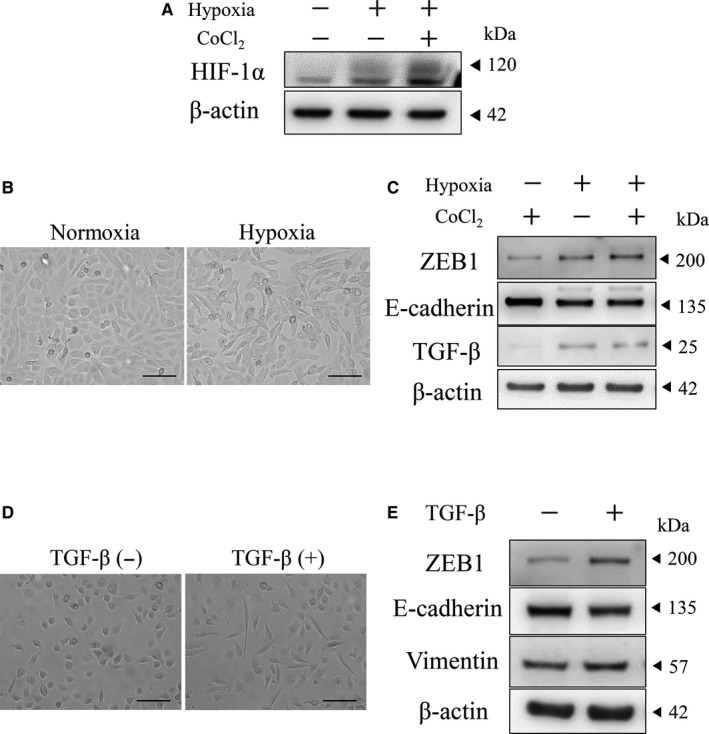
Hypoxia induced HIF‐1α and ZEB1 expressions and EMT in AM‐1 cells. A, Western blots of HIF‐1α in normoxia (hypoxia: −) or hypoxia (hypoxia: +) condition with or without 100 μmol/L CoCl_2_. Molecular weight is pointed by an arrowhead. B, Morphological changes of AM‐1 cells in normoxia (a) and hypoxia (b) condition. Scale bars: 100 μm. C, Western blots of ZEB1, E‐cadherin and TGF‐β in hypoxia (−) and hypoxia (+) condition with or without 100 μmol/L CoCl_2_. Molecular weight is pointed by an arrowhead. β‐actin; internal control. D, Morphological changes of AM‐1 cells in control (a) and TGF‐β treatment (b). Scale bars: 100 μm. E, Western blots of ZEB1, E‐cadherin and vimentin with or without TGF‐β. Molecular weight is pointed by an arrowhead. β‐actin; internal control

To clarify the role of HIF‐1α in ABs and ACs, we performed the in vitro hypoxic culture using a human ameloblastoma cell line, AM‐1. In the hypoxic condition, the increase of HIF‐1α expression was observed in Western blotting (Figure 3A). It is well known that CoCl_2_ inhibits HIF‐1α degradation. To elucidate roles of HIF‐1α in AM‐1, we examined the gain of HIF‐1α function by adding CoCl_2_ in the AM‐1 culture system. As a result, further accumulation of HIF‐1α was seen in CoCl_2_ treatment in the hypoxic condition (Figure 3A). In this hypoxic condition, AM‐1 changed the morphology from the oval or round appearance (Figure 3B; a) to the spindle‐shaped one (Figure 3B; b). Interestingly, the same morphological change was seen in the in vitro EMT induction stimulated by transforming growth factor‐β (TGF‐β) (Figure 3D). In addition, AM‐1 cells with hypoxic culture showed increase of ZEB1 and TGF‐β and decrease of E‐cadherin expression in Western blotting (Figure 3C). These alterations of protein expressions were similar to those recognized in the EMT induction experiment by TGF‐β (Figure 3E). In addition, when we suppressed ZEB1 protein expression using siRNA in AM‐1 cells, TGF‐β‐induced EMT was inhibited, which revealed that ZEB1 expression was downstream of TGF‐β signaling (Supporting Information 2). These results indicated that hypoxia‐induced HIF‐1α and ZEB1 expressions and following EMT‐like morphological changes depending on the TGF‐β expression in AM‐1 cells.

### 3.4 Tranilast inhibited hypoxia‐induced EMT and migration cell ability in AM‐1 cells

**Figure 4:**
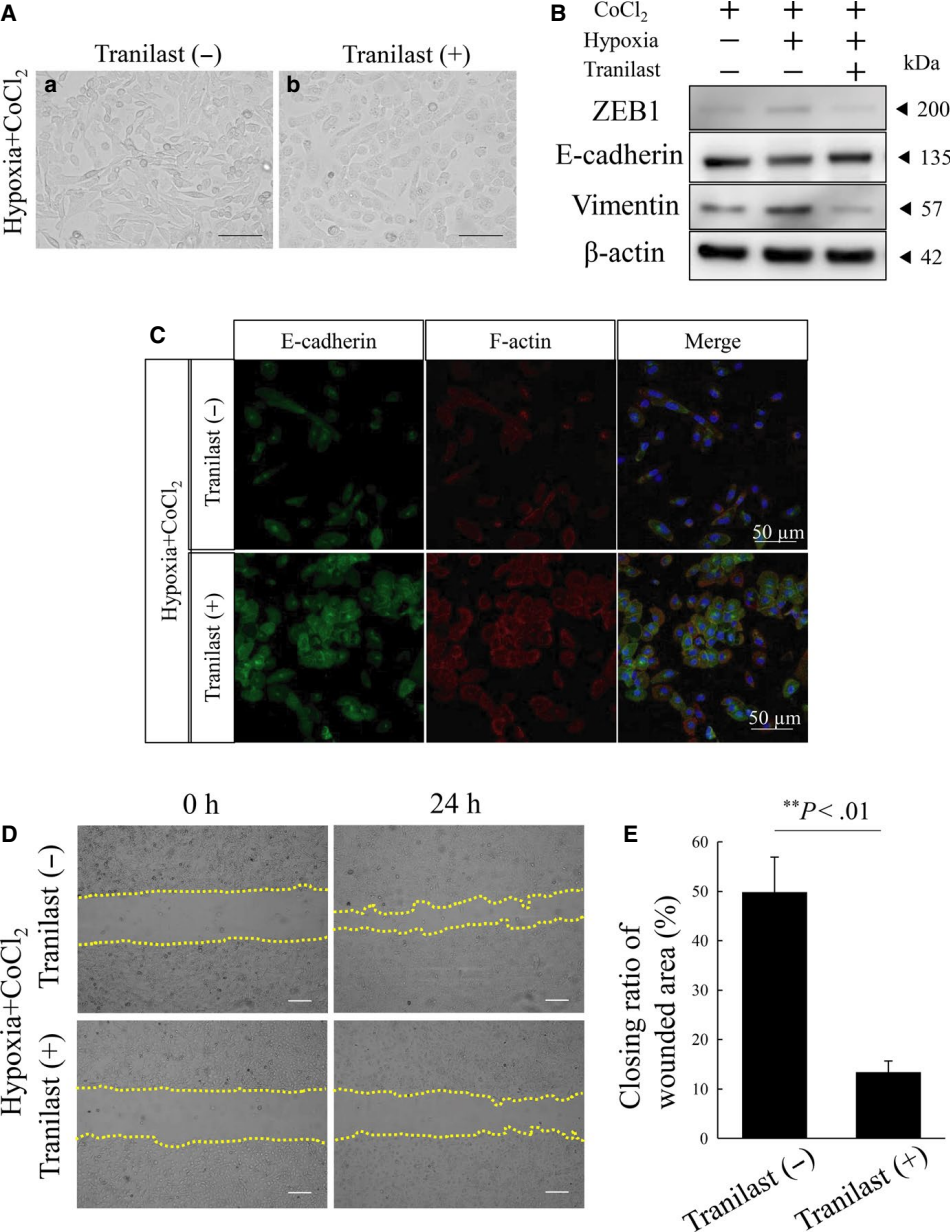
Tranilast inhibits hypoxia‐induced EMT and migration activity in AM‐1 cells. A, Tranilast inhibited morphological changes recognized in EMT induction. Panels are hypoxic culture of AM‐1 with 100 μmol/L CoCl_2_ untreated (a) and treated (b) with 100 μmol/L tranilast_._ Scale bars: 100 μm. B, Western blots of ZEB1, E‐cadherin and vimentin in normoxia or hypoxia with or without tranilast. Molecular weight is pointed by an arrowhead. C, Immunocytostaining of E‐cadherin and F‐actin in hypoxic culture with or without tranilast. Scale bars: 50 μm. D, Wound healing assay to evaluate the migration ability of AM‐1 cells in hypoxia with (lower panels) or without (upper panels) tranilast. The closing pattern of wounded area is displayed in panels by dotted lines. The culture is stopped after 24 h (each right column) from the start 0 h (each left column). E. Bars indicate the closing ratio of wounded area in hypoxia with (right bar) or without (left bar) tranilast. Statistical significance was set as **P < .01

We found that TGF‐β was induced by the HIF‐1α and ZEB1 induction in the hypoxic state and then played an important role in the EMT induction in AM‐1 cells. Based on this finding, we next tried to confirm if TGF‐β induced by HIF‐1α and ZEB1 in hypoxia critically played an important role in the induction of EMT by inhibiting this pathway using tranilast. Tranilast; *N*‐(3′,4′‐dimethoxycinnamoyl) anthranilic acid (*N*‐5′), one of the antiallergic drags, is known as a TGF‐β inhibitor.^18^ Predictably, tranilast inhibited hypoxia‐induced spindle‐shaped morphological changes (Figure 4A). Western blotting analysis also indicated that tranilast inhibited ZEB1 and vimentin inductions and E‐cadherin reduction in hypoxia (Figure 4B). Immunocytochemistry revealed that E‐cadherin expression was repressed and translocated from cell surface to cytosol in AM‐1 cells by tranilast treatment in hypoxia with CoCl_2_ culture condition (Figure 4C). In the wound healing assays, CR was significantly decreased resulting in the inhibition of the AM‐1 cell migration ability by tranilast treatment in hypoxia with CoCl_2_ culture condition (Figure 4D, 4). These results revealed that TGF‐β induced by the HIF‐1α and ZEB1 induction in hypoxia played an important role in the induction of EMT and tranilast treatment might be a potentially useful therapeutic approach to prevent the malignant transformation of AB or progression of AC in hypoxic tumor microenvironments.

## 4 DISCUSSION

In this report, we aimed to clarify the mechanisms of malignant transformation of AB or AC carcinogenesis. At the first step of this experiment by the DNA microarray and scatterplot analyses against AC and preexisting AB, we focused on two genes, *HIF1A* and *ZEB1*, as favorable genes for our aim. Next, by the semiquantitative immunohistochemical analyses, we revealed that the expressions of these two proteins were both significantly higher in ACs than in ABs. In addition, we immunohistochemically recognized the tendency of co‐expression of HIF‐1α and ZEB1 in the nuclei of AC cells especially in the regions showing prominent invasive or aggressive growth of AC cells with a loss of cell‐cell adhesion or individual growth appearances. At last, we substantiated the evidence in vitro that hypoxia‐induced HIF‐1α and ZEB1 expressions and EMT‐like morphological changes in AM‐1 cells, then the TGF‐β inhibition assay using tranilast revealed that these changes were dependent on the TGF‐β expression induced by HIF‐1α and ZEB1 expressions. Finally, we concluded that hypoxia‐induced HIF‐1α and ZEB1 are critical for the malignant transformation of AB via TGF‐β‐dependent EMT.

AC is a rare and a malignant odontogenic tumor showing an aggressive clinical behavior and a poor prognosis. Nonetheless, there have been currently no specific markers for ACs other than Ki‐67 labeling index score which was reported to be a potential indicator of AC.^19^ Thus, the discovery of some other useful biomarkers for ACs has been strongly required not only for making a definite pathological diagnosis but also for developing a new clinical therapy for the prevention of ACs. In this report, we revealed that HIF‐1α and ZEB1 in the hypoxic tumor microenvironments would be new potential biomarkers for ACs and then the secondary induced TGF‐β to which EMT induction was dependent would be a critical clinical target for the AC therapy.

In the carcinogenesis, the accumulation of driver gene mutations was regarded as a main factor in various carcinomas.^6^, ^20^, ^21^ In the ameloblastoma, *SMO* and *BRAF* mutations were frequently seen.^22^ Interestingly, *BRAF* mutation was also reported in AC.^23^ Nobusawa et al reported a case of AC which developed in preexisting AB with a mutation of p53 gene.^24^ Thus, it is suggested that some driver gene mutations accumulated in AB contribute to AC carcinogenesis. In addition to the driver mutation, alteration of tumor microenvironments was critical for cancer progression.^6^, ^7^ In these environments, HIF‐1α was known as a master regulator to adapt hypoxic condition.^25^ It is also well known that HIF‐1α was upregulated in many types of cancers.^9^, ^10^, ^26^ From these previous reports and our results, HIF‐1α in the hypoxic condition of tumor microenvironments would play a role as one of the major triggers of malignant transformation from AB to AC. However, the detail has not been clarified yet which driver mutation upregulates AB proliferation and induces activation of HIF‐1α following local hypoxia.

It is well known that TGF‐β was secreted by stromal fibroblasts, macrophages, endothelial cells, and tumor cells in tumor microenvironments 27, 28, 29 and was a pivotal inducer of EMT both in the fetal development and in the cancer progression.^30^, ^31^ In addition, TGF‐β contributed not only to tumor cell invasion but also to heterogeneities in cancer stem cells.^32^ McLean‐Holden et al recently reported three cases of AC with EMT features.^33^ Moreover, TGF‐β was upregulated by HIF‐1α in gastric cancer, breast cancer, and dermal fibrosis.^34^, ^35^, ^36^ Together with these previous reports, our data from the hypoxic culture of AM‐1 revealed that hypoxia‐induced HIF‐1α and subsequently TGF‐β expressions in AB, resulting in the induction of EMT and the potentiation of tumor cell progression or invasion.

Recently, attention has been much payed to the ability of neoplastic epithelial cells to re‐enter the stem cell state, and the generation of new cancer stem cells (CSCs) from non‐CSC populations was considered one of the significant causative mechanisms for cancer cells to obtain much aggressiveness. In addition, the cell plasticity switching from a non‐CSC to CSC state was dependent on ZEB1, a critical regulator of EMT. Namely non‐CSCs maintained the ZEB1 promoter in a bivalent chromatin configuration to readily respond to microenvironmental signals such as TGF‐β. The ZEB1 promoter responding such signals converted from a bivalent to active chromatin configuration to increase ZEB1 transcription and then non‐CSCs subsequently entered the CSC state.^37^ In some reports, HIF‐1α promoted ZEB1 expression and EMT in hypoxia and this HIF‐1α/ZEB1 axis contributed the enhancement of cancer cell aggressiveness in such migration and invasion or distant metastasis in bladder cancer and glioblastoma.^38^, ^39^ In our study, HIF‐1α and ZEB1 were both significantly and relatively upregulated in AC than in AB by the DNA microarray analysis. Thus, the HIF‐1α/ZEB1 axis in hypoxia might also contribute to the malignant transformation of AB or the enhancement of AC cell aggressiveness and become a critical therapeutic target in AC for improving the prognosis.

Tranilast, an analog of a tryptophan metabolite, was first reported in 1976 by Koda et al as an antiallergic agent and used in the treatment of inflammatory diseases such as bronchial asthma, keloids, and hypertrophic scars.^18^ In 1987, it was reported that tranilast inhibited the proliferation of fibroblasts and selectively suppressed collagen deposition; then, tranilast was viewed as a novel antiproliferative agent.^40^ Furthermore, it became apparent that in addition to normal cells, tranilast effectively inhibited the proliferation of several tumor cells such as gastric,^41^ pancreatic,^42^ prostate, 43 and breast 44 cancer cells, malignant glioma cells 45, and squamous cell carcinoma.^46^ Thus, tranilast is now also considered to be an antitumor agent. Recently, the major target for tranilast was reported to be the suppression of the TGF‐β signaling pathway.^18^ In carcinogenesis, TGF‐β plays a paradoxical role in each different stage. Namely TGF‐β suppresses the progression of early lesions, but later cancer cells subsequently produce TGF‐β and at this stage TGF‐β contributes to tumor progression. For this latter effect, it was also reported that tranilast inhibited the expression and/or secretion of TGF‐β from cancer cells or stromal cells.^43^ In our in vitro culture study using AM‐1 cells, tranilast downregulated the migration activity by inhibiting ZEB1 expression and EMT induction. These data are also compatible with those in the report that tranilast inhibited the expression of genes related to EMT by the suppression of TGF‐β signaling pathway.^47^ Thus, tranilast might also become a potential therapeutic agent for preventing the malignant transformation of AB and AC progression.

In summary, we here clarified that HIF‐1α and ZEB1 were significantly upregulated in AC and were also highly expressed in AC cells. Hypoxia induced HIF‐1α and ZEB1 expressions and EMT in AM‐1 cells. These inductions were inhibited by the inhibition of TGF‐β signaling pathway using tranilast. Then, the migration activity of AM‐1 cells was also inhibited by the suppression of TGF‐β signaling pathway with tranilast treatment. Finally, we concluded that hypoxia‐induced HIF‐1α and ZEB1 are critical for the malignant transformation of AB via TGF‐β‐dependent EMT. Then, tranilast might be effective for preventing the malignant transformation of AB and AC progression.

## CONFLICT OF INTEREST

The authors have no conflict of interest to declare.
