# Supplementary figures and images for: Hypoxia‐induced HIF‐1α and ZEB1 are critical for the malignant transformation of ameloblastoma via TGF‐β‐dependent EMT
Source: Cancer Med. 2019 Nov 1;8(18):7822–32. doi: 10.1002/cam4.2667 (PMC6912026; doi:10.1002/cam4.2667)

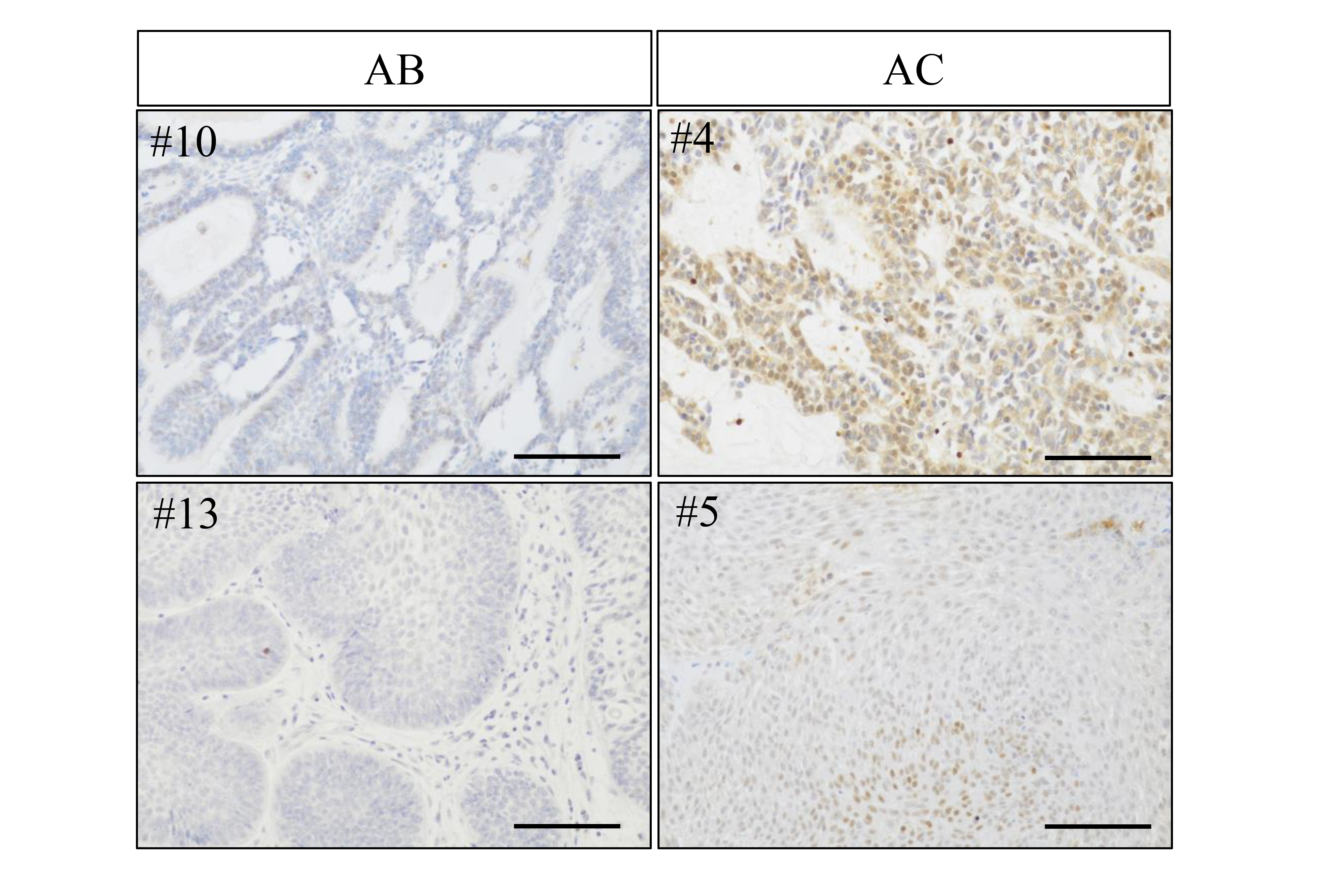

Supplement: Supplementary file 1 [file CAM4-8-7822-s001.tif]

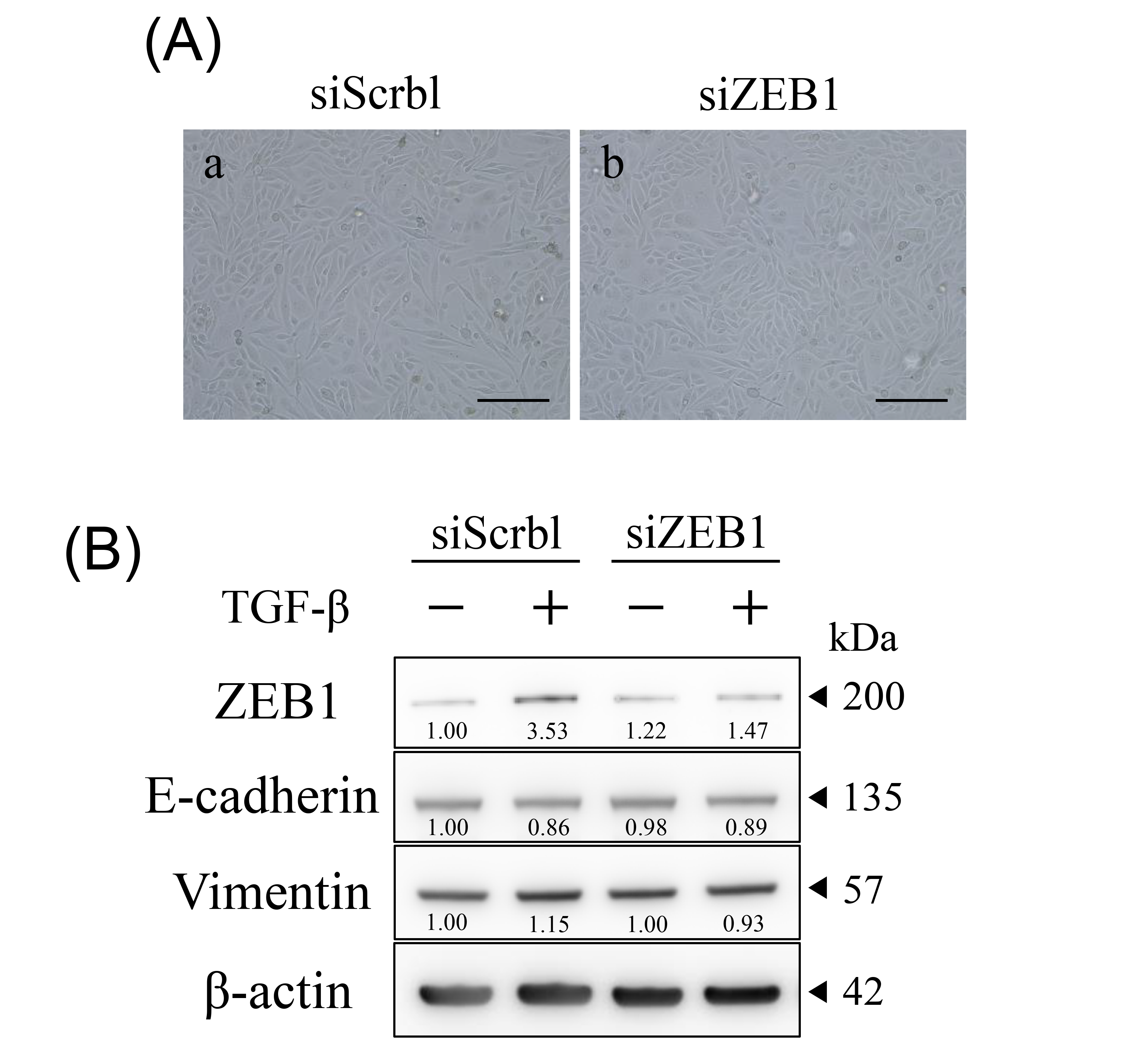

Supplement: Supplementary file 2 [file CAM4-8-7822-s002.tif]
